# Prof. Flint’s Doctrine of the Self-Limitation of Phthisis

**Published:** 1890-11

**Authors:** William Porter

**Affiliations:** Professor of Laryngology and Diseases of the Chest, in the St. Louis College of Physicians and Surgeons; Fellow of the American Laryngological Association; Ex-President of the Mississippi Valley Medical Association


### PROFESSOR FLINT'S DOCTRINE OF THE SELF-LIMITATION OF PHTHISIS.*

BY WILLIAM PORTER, M. D.

Professor of Laryngology and Diseases of the Chest, in the St. Louis College of Physicians and Surgeons; Fellow of the American Laryngolcgical Association; Ex-President of the Mississippi Valley Medical Association.

Sometime before his death Prof. Flint promulgated the doctrine of the self-limitation of phthisis and presented it with all of his well known power and great ability to the profession. This very interesting proposition was at the time the subject of free

*Read at the 16th annual meeting of the Mississippi Valley Medical Association at Louisville, Oct 8th, 1890.

debate in various medical societies, and at a former meeting of the Association I had the honor to partially discuss the position taken by this learned teacher and writer.

The recent years have been full of the wonderful results of the study of pulmonary disease and bacteriologic research and the possibility of a positive diagnosis has overshadowed the equally interesting question of prognosis. That Prof. Flint's arguments, however, have not been lost to the profession, is evidenced by the repeated references to it in recent current medical literature, and many are ready to say with Prof. Stephen Burt, of New York, that "not a few cases of phthisis have a self-limitation." This author in a valuable paper entitled "Pulmonary Consumption in the Light of Modern Research," read before the New York Academy of Medicine, and published in the *Medical Record*, April 12th, 1890, takes the tenable position that "phthisis is an infectious disease, only the soil must be fertile or the bacteri will not take root and grow; that the inheritance of the affection is simply the descent of the degraded cells presenting a vulnerable point for the vagrant germs; that all specific treatment is futile; and though persistent destruction of the infectious matter is our best means of prophylaxis, yet to restore the vitality of the lung tissue is as important as to destroy the tubercle bacilli." Following this comes his affirmation of the possibility of the self-limitation of phthisis. In Pepper's elaborate System of Medicine, Vol. III., p. 393, Flint again affirms his statement that in certain cases of phthisis "the disease may be said to be self-limited."

It is a just inference that this idea has been well received by many excellent physicians and that it has made a deep impression upon the mind of the profession, not more on account of its great champion than because of its plausibility and desirability, for truly as one author says, "it is a comforting thought to the afflicted" and we would add—to the physician also.

I can bring no more interesting question before you; it touches us at all points where we attempt to save life from this dread disease, and it is inseparably connected with our opinion regarding the future of a large proportion of our patients. It is right therefore that we should have some fixed idea as to the existence of self-limitation of phthisis and its value in any given case or series of cases.

After having carefully examined the facts cited in support of the proposition, I have no hesitation in distinctly asserting that I find no sufficient evidence to warrant us in accepting the statement that phthisis is self-limiting, or that the element of self-limitation has a decided influence upon the result in any given case. I do not mean that all cases of phthisis necessarily die from this disease, but I do mean that where phthisis is firmly established, there is nothing in the nature of the disease itself that indicates in any stage a fixed boundary—a line of demarcation as it were, but rather that all of its tendencies are progressive and downward.

It is well that there should be in all debate a clear understanding as to the meaning of the terms employed, and at the outset I am perfectly willing to accept Flint's own definition not only of the term "self-limiting" but also of the word "phthisis."

Permit me then to quote in full his definitions as given in his argument: "A disease is self-limited when it ends in recovery irrespective of extrinsic influences derived from either hygiene or therapeutics. A patient, whatever be the disease, who recovers without any potential remedies or measure of treatment having been employed, and where there has been no material change in any of the circumstances pertaining to daily life, owes the recovery exclusively to self-limitation." To this we readily agree.

He then defines the terms pneumonic phthisis and pulmonary consumption, and says, "I shall consider the terms as applicable to all cases of phthisical disease exclusive of acute tuberculosis and interstitial (fibroid) pneumonia." That there may be no mistake as to his use of words, I may add that he uses the terms phthisis, pulmonary tuberculosis and consumption in the same sense (Prac. Med., p. 271.) Using then our author's own language, the question is —Is there in phthisis, or if you please pulmonary tuberculosis, a tendency to recovery without extrinsic influence derived from either hygiene or therapeutics ?

Although as early as 1835, Jacob Bigelow, in a paper which Prof. Flint justly calls remarkable, read before the Massachusetts Medical Society, applied the term self-limited to certain diseases, it was not until 1858 that the latter advanced the idea of self-limitation as applied to phthisis, (Am. Jour. Med. Sciences, 1858.) Twenty years afterward the question was ably re-opened by him in the paper to which reference has been made, and his position virtually accepted as proven.

The deductions in this important essay are based upon clinical evidence rather than argued from a pathological standpoint, and if you will bear with me I will first present an analysis which I have made of the cases which are cited by Prof. Flint, and afterward endeavor to show that the pathology of phthisis, so far as we yet understand it, is opposed to the doctrine of self-limitation.

In the first place the argument which Prof. Flint advances is founded upon the deductions made from the history of 670 cases of phthisis. Fortunately these cases are all on record, and I shall not only accept the record of each case without question, but follow his own selection, though to very different conclusions.

Among these 670 cases, there were seventy-five in which either recovery took place or the disease became latent. It is therefore in these seventy-five cases we have to find the proof of self-limitation, and by these must the proposition stand or fall. I must beg you to bear in mind as we go along the definition that "a disease is self-limited when it ends in recovery irrespective of extrinsic influences derived from either hygiene or therapeutics."

We find that in thirty-one of these seventy-five cases the statement is merely that "the disease ceased to progress for at least several months, and in the majority of cases for several years. "By reference to the record we find that the last examination of each gave evidence that the disease was still present—latent in some, as may occur in phthisis, but not self-limited; for 'a disease is self-limited when it ends in recovery,' etc., and these had not recovered." As, according to our author's own definition, thirty-one of these cases have no definite bearing upon the point in question, we are restricted to the study of the remaining forty-four.

As self-limitation is, independent "of extrinsic influence, derived from either hygiene or therapeutics," we at once decline the evidence of twenty-one of the forty-four cases, for in all of these pertinent and generally persistent treatment was pursued. Moreover three of these cases subsequently proved fatal, and the last examination showed that at least a third of them had still physical signs of phthisis. We object to these twenty-one histories as not pertinent. The interest now centers in but twenty-three. "In fifteen of the cases hygienic measures constituted the treatment;" but these measures were of such a character as would lead us to hope for favorable results, viz., change of business, out of door life, rest, sea voyages, change of climate, etc. These are potential aids, for as Flint says (Prac. Med., p. 290), "out of door life is of all measures most important." Now, to prove a disease self-limiting, we must eliminate whatever can be reasonably traced to "either hygiene or therapeutics." These fifteen cases were given the advantage of favorable hygienic conditions, and who shall say they would have recovered without these conditions? Having made use of that remedy which of all others has been found efficacious in phthisis, these fifteen cases are certainly not examples of self-limitation.

I have refrained from occupying your time with the details of the author's cases which, in accordance with his own definition of limitation, have been refused as evidence. The history of these is fully given in his work on phthisis, and I have endeavored to deal fairly and justly with the record.

Let us now apply the test to eight cases which alone remain. These are numbered 1, 4, 7, 8, 14, 20, 23 and 24 (Phthisis, p. 187 *et seq.*), and are the only ones of which the author says "there was no medicinal treatment of importance, and no material change in the habits of life, the recovery taking place purely from an intrinsic tendency."

Case I. is that of a farmer who, having, in the winter of 1842-43, expectorated what were thought to be pulmonary calculi, was examined in June, 1843; "the only physical sign noted was feebleness of the respiratory murmur." He was in excellent health thirteen years afterward. "Prior to the development of the disease, the patient had worked very hard on a farm. He left home for several weeks, and after relinquishing severe labor, engaged in buying and selling new lands in Illinois, a business which required much out of door life." Excellent treatment!

Case IV. A physician aged twenty-eight, had hæmoptysis in October, 1852, and again in January and May, 1853. In May, 1853, he had slight dullness at the right summit with weakened respiratory murmur and crackling, which accompanied inspiration and expiration. In September, 1854, he reported himself well, "a year after recovery;" i. e., his recovery must have dated from September, 1853, five months only after the above symptoms were noted. However, we find that in September, 1854, there was still a dullness at the right summit, and the respiratory murmur was feeble."

Case VII. is that of a constable, examined in April, 1856. Six years before he had had a hemorrhage, and shortly afterwards recurrent hæmoptysis for ten days. During the following year Prof. Flint met this man from time to time on the street, and he seemed to be in good health. As this man evidently had phthisis for six years prior to examination, what reason is there, in the absence of a later examination, to suppose that he had entirely recovered during one succeeding year? The statement that he seemed well will apply, at times, to many cases of chronic phthisis. "There is no further record of this case. The treatment consisted of cod liver oil for six weeks, generous living, the use of malt liquors, and out-of-door life." Very good intrinsic influences we take it.

Case XIV. A physician, who had cough, hæmoptysis and slight loss of flesh, was examined October, 1857, and found to have evidence of phthisis at the left apex. Five years later Dr. Flint saw him in apparently good health. He had been drinking beer, living generously, with an abundance of exercise out of doors. It seems that in this case and in number 4, no medicinal agent was used, though the patients were physicians. They both had the influence of riding and driving in the open air while engaged in country practice.

In Case XXIV. we find that the patient consulted Dr. Flint by letter in 1859; was relieved of part of her duties as a teacher; took more out of door exercise ; traveled in the summer. In 1862 had abnormal dullness, feeble respiration, and increase of resonance and whisper at the right summit. Afterwards traveled in Europe; in 1858 had hæmoptysis; in 1869 increase of symptoms, and she died in the spring of 1871. Did this patient recover, and that without hygienic change of condition?

The three remaining cases do, so far as recorded, seem to be instances of recovery from phthisis without medical or hygienic agency.

Number VIII. was a clerk, examined in August, 1856, having had cough two months previously and hemorrhage a week before. There was a dullness and feeble respiration at the right apex and sub-crepitant rules on both sides. In October, 1856, he was reported well, and was in good health in 1871. The thought is at once suggested, why did he, being examined at a time of greatest danger, not have medicinal treatment. The case is, however, one of great interest, and is certainly an exception to the general rule, as the disease appeared, progressed and abated within a period of five months.

The other two cases, XX., and XXIII., furnish the best evidence in favor of self-limitation, though the record is very short. Two sisters, whose parents, three sisters and two brothers had died of phthisis, were found, one with disease of the left apex, the other of the right. No remedy of importance was given, or change made in the habits of life. Both were well fifteen years afterward. Again the question may be asked, why was not some form of treatment or change of condition ordered in these cases, as " no effort had been spared to save the lives of their sisters and brothers; traveling, change of climate, together with remedies having been resorted to in vain, although, perhaps, with the effect of retarding the progress of the disease." With this, however, we have nothing to do. The record is that these two sisters, for whom nothing was done, alone recovered. Granting that these two cases, and possibly the preceding one show evidence of self-limitation, yet they are but three out of 670, and we again quote from Prof. Flint's paper, "Self-limitation cannot be inferred from a single case or a very few cases."

The clinical evidence cited by Prof. Flint certainly does not prove the doctrine of self-limitation according to his own definitions, for we find that in the large experience of its able advocate, among hundreds of cases as recorded by himself, that the argument is sustained by few, and in all fairness we confess a doubt as to the pertinence and value of most of these.

Is not this conclusion in accord with our own experience? What physician to-day expects that in a given number of cases of phthisis as they come to him, any small proportion will recover without the help of medication or change in hygiene or environment? Or to put the question more directly, who has so much faith in the doctrine of self-limitation that he would trust it in the slightest degree in planning for the future good of his patients?

So much as we know of the true pathology of phthisis is opposed to the doctrine of self-limitation. In the constitutional predisposition or the general morbid condition which Flint speaks of as the essential disease, we now recognize only that degeneration of the body in whole or in part, which favors the reception and development of the specific factor of tuberculosis. So long as no bacilli are found, the disease cannot be proven to be tubercular. The tubercular cachexia is a misnomer, except so far as it is limited to conditions which favor the invasion of the essential germ.

In the ordinary case of tubercular phthisis we now know that we have not only the constitutional fault, characterized, as Sir Andrew Clark has it, "by a progression of symptoms with an ulcerative or suppurative destruction of a more or less circumscribed, non-malignant deposit in the lung," but in addition, we have a specific morbid change, the result of a specific cause. I need not weary you with a discussion of the exact relation of the bacillus to tuberculosis. Whether the bacilli are the cause or the result of the rapid disintegration is not the question in point.

Whether these or their ptomaines or both are the active factors may some day be fully decided. This much we know, that where the changes have begun which result in those morbid products in which the tubercle bacilli are found, we have to deal with an active, relentless, progressive foe to human life.

Let us but remember that the bacillus readily enters a mucous membrane denuded of its epithelium and passes down to the lymphatics and vessel walls; that, according to Metschnikoff and Naegeli, it inaugurates a struggle with and rapidly destroys feeble organic cells, and we must forever abandon all preconceived ideas of the self-limitation of phthisis.

Our author says "there should be no room to doubt as to the accuracy of the diagnosis," and the researches of the bacteriologist have placed within our reach, the essential element in the diagnosis of tuberculosis, but the knowledge of the existence of this element is directly opposed to the docrine of limitation without extrinsic aid.

Therefore clinically and pathologically we must have further evidence of self-limitation. I grant you that well proven cases get well. It must be admitted that treatment has in some instances been successful; that resistance has been made to the cell invasion of the bacilli and that the general systemic fault has been corrected. My position is, however, that in every such case, there has been some helpful treatments administered, some beneficial hygienic or climatic change accomplished.

Our deductions must, therefore, be:

1st. That there is no sufficient clinical evidence to warrant us in believing that by self-limitation, as defined by Prof. Flint, pulmonary phthisis may end in recovery.

2d. The pathology of phthisis is equally opposed to the proposition.

3d. Although phthisis is not self-limited, yet limitation is pos sible through "extrinsic influence derived from hygiene and therapeutics."

One word in conclusion. To no one does the medical profession of America owe more than it does to the memory of Dr. Flint. Though dead, his words live. With reverent hands would we lift the record of his work and in the same fairness which he admired and practiced, would we examine his teachings. In this city which he loved so well and in which was once his home, no words are needed to recall his greatness.

May the analysis of his arguments which we have made, and even the widely different conclusions which we have reached, be counted an honest tribute to the great influence of him whose lips are forever sealed.

